# Validation of dose distribution for liver tumors treated with real-time-image gated spot-scanning proton therapy by log data based dose reconstruction

**DOI:** 10.1093/jrr/rrab024

**Published:** 2021-05-05

**Authors:** Takahiro Yamada, Seishin Takao, Hidenori Koyano, Hideaki Nihongi, Yusuke Fujii, Shusuke Hirayama, Naoki Miyamoto, Taeko Matsuura, Kikuo Umegaki, Norio Katoh, Isao Yokota, Hiroki Shirato, Shinichi Shimizu

**Affiliations:** Hitachi Ltd. 1-1 7-chome, Oomika-cho, Hitachi-shi, Ibaraki 319-1292, Japan; Graduate School of Biomedical Science and Engineering, Hokkaido University, North15 West7, Kita-ku, Sapporo, Hokkaido 060-8638, Japan; Department of Medical Physics, Hokkaido University Hospital, North14 West5, Kita-ku, Sapporo, Hokkaido 060-8638, Japan; Division of Quantum Science and Engineering, Faculty of Engineering, Hokkaido University, North13 West8, Kita-ku, Sapporo, Hokkaido 060-8638, Japan; Global Station of Quantum Medical Science and Engineering, Global Institution for Collaborative Research and Education, Hokkaido University, North15 West7, Kita-ku, Sapporo, Hokkaido 060-8638, Japan; Department of Medical Physics, Graduate School of Medicine, Hokkaido University, North15 West7, Kita-ku, Sapporo, Hokkaido 060-8638, Japan; Hitachi Ltd. 1-1 7-chome, Oomika-cho, Hitachi-shi, Ibaraki 319-1292, Japan; Hitachi Ltd. 1-1 7-chome, Oomika-cho, Hitachi-shi, Ibaraki 319-1292, Japan; Hitachi Ltd. 1-1 7-chome, Oomika-cho, Hitachi-shi, Ibaraki 319-1292, Japan; Graduate School of Biomedical Science and Engineering, Hokkaido University, North15 West7, Kita-ku, Sapporo, Hokkaido 060-8638, Japan; Department of Medical Physics, Hokkaido University Hospital, North14 West5, Kita-ku, Sapporo, Hokkaido 060-8638, Japan; Division of Quantum Science and Engineering, Faculty of Engineering, Hokkaido University, North13 West8, Kita-ku, Sapporo, Hokkaido 060-8638, Japan; Global Station of Quantum Medical Science and Engineering, Global Institution for Collaborative Research and Education, Hokkaido University, North15 West7, Kita-ku, Sapporo, Hokkaido 060-8638, Japan; Department of Medical Physics, Hokkaido University Hospital, North14 West5, Kita-ku, Sapporo, Hokkaido 060-8638, Japan; Division of Quantum Science and Engineering, Faculty of Engineering, Hokkaido University, North13 West8, Kita-ku, Sapporo, Hokkaido 060-8638, Japan; Global Station of Quantum Medical Science and Engineering, Global Institution for Collaborative Research and Education, Hokkaido University, North15 West7, Kita-ku, Sapporo, Hokkaido 060-8638, Japan; Department of Medical Physics, Hokkaido University Hospital, North14 West5, Kita-ku, Sapporo, Hokkaido 060-8638, Japan; Division of Quantum Science and Engineering, Faculty of Engineering, Hokkaido University, North13 West8, Kita-ku, Sapporo, Hokkaido 060-8638, Japan; Global Station of Quantum Medical Science and Engineering, Global Institution for Collaborative Research and Education, Hokkaido University, North15 West7, Kita-ku, Sapporo, Hokkaido 060-8638, Japan; Global Station of Quantum Medical Science and Engineering, Global Institution for Collaborative Research and Education, Hokkaido University, North15 West7, Kita-ku, Sapporo, Hokkaido 060-8638, Japan; Department of Therapeutic Radiology, Faculty of Medicine, Hokkaido University, North15 West7, Kita-ku, Sapporo, Hokkaido 060-8638, Japan; Department of Biostatistics, Graduate School of Medicine, Hokkaido University, North15 West7, Kita-ku, Sapporo, Hokkaido 060-8638, Japan; Global Station of Quantum Medical Science and Engineering, Global Institution for Collaborative Research and Education, Hokkaido University, North15 West7, Kita-ku, Sapporo, Hokkaido 060-8638, Japan; Department of Proton Beam Therapy, Faculty of Medicine, Hokkaido University, North15 West7, Kita-ku, Sapporo, Hokkaido 060-8638, Japan; Department of Medical Physics, Hokkaido University Hospital, North14 West5, Kita-ku, Sapporo, Hokkaido 060-8638, Japan; Global Station of Quantum Medical Science and Engineering, Global Institution for Collaborative Research and Education, Hokkaido University, North15 West7, Kita-ku, Sapporo, Hokkaido 060-8638, Japan; Department of Radiation Medical Science and Engineering, Faculty of Medicine, Hokkaido University, North15 West7, Kita-ku, Sapporo, Hokkaido 060-8638, Japan

**Keywords:** proton therapy, spot scanning, log data, dose reconstruction, motion management

## Abstract

In spot scanning proton therapy (SSPT), the spot position relative to the target may fluctuate through tumor motion even when gating the radiation by utilizing a fiducial marker. We have established a procedure that evaluates the delivered dose distribution by utilizing log data on tumor motion and spot information. The purpose of this study is to show the reliability of the dose distributions for liver tumors treated with real-time-image gated SSPT (RGPT). In the evaluation procedure, the delivered spot information and the marker position are synchronized on the basis of log data on the timing of the spot irradiation and fluoroscopic X-ray irradiation. Then a treatment planning system reconstructs the delivered dose distribution. Dose distributions accumulated for all fractions were reconstructed for eight liver cases. The log data were acquired in all 168 fractions for all eight cases. The evaluation was performed for the values of maximum dose, minimum dose, D99, and D5–D95 for the clinical target volumes (CTVs) and mean liver dose (MLD) scaled by the prescribed dose. These dosimetric parameters were statistically compared between the planned dose distribution and the reconstructed dose distribution. The mean difference of the maximum dose was 1.3% (95% confidence interval [CI]: 0.6%—2.1%). Regarding the minimum dose, the mean difference was 0.1% (95% CI: −0.5%—0.7%). The mean differences of D99, D5–D95 and MLD were below 1%. The reliability of dose distributions for liver tumors treated with RGPT-SSPT was shown by the evaluation of the accumulated dose distributions.

## INTRODUCTION

The proton spot scanning technique [1–3] increases conformity to the target volume and modulates the dose more flexibly than the passive scattering technique. Proton spot scanning delivers the proton dose distribution as a set of discrete and narrow pencil beams such that the target volume is covered in Bragg peaks depending on the depth. In order to deliver a conformal 3D dose distribution, several thousand such beams, each with different energies and positions, are required. In spot scanning, however, the dose distribution may deteriorate under organ motions, such as respiratory or cardiac motions.

Real-time 4-dimensional radiotherapy, which includes beam gating [4–7] and beam tracking [8, 9], has been used in photon therapy to mitigate the dosimetric impacts of target motion. Gating [10–13] and tracking techniques [14, 15] using external surrogates have also been reported in particle therapy. However, the internal organ motions and the external surrogate signals of abdominal motion are not necessarily correlated during treatment [16, 17]. The real-time-image gated spot-scanning proton beam therapy (RGPT) system in our institute is able to perform gated irradiation based on real-time monitoring utilizing internal fiducial markers [18–21]. In RGPT, the proton beam is gated on the basis of the marker position calculated from fluoroscopic X-ray images acquired at a rate of 30 Hz. Gated irradiation with markerless tumor tracking has been used in carbon ion therapy [22]. By gated irradiation, the dose variation is mitigated, but the dosimetric impacts of the residual motion still need to be considered.

For patient-specific quality assurance, dose validation methods utilizing machine log files have been reported. Li *et al.* [23] and Zhu *et al.* [24] calculated dose distributions in patient’s computed tomography (CT) images based on the spot information on the delivered dose and spot positions acquired from log data of treatment machines. Meier *et al.* [25] and Scandurra *et al.* [26] evaluated the delivered dose distribution by using log-data-based calculations independent of the treatment planning system.

However, for evaluating the dose distributions in the case of treatment of moving organs, not only errors in the beam position and delivered dose, but also organ motion needs to be taken into consideration. Colvill *et al.* [27] developed a method of motion-including dose reconstruction that uses target motion trajectory. In this method, the dose distribution is calculated with simulated timing data of beam delivery under the assumption of accelerator operation, instead of utilizing log data.

For a more precise evaluation, the dose distribution should be calculated on the basis of log data of tumor motion and log data of beam delivery that include timing information of the spot irradiations. Furthermore, these log data should be synchronized to within a few milliseconds accuracy in order to suppress a spot position error due to synchronization error to about 0.1 mm. We established a procedure that evaluates the delivered dose distribution on the basis of log data on tumor motion and beam delivery [28]. Prior to this study, the accuracy of the log data was verified. It was confirmed that the synchronization accuracy was less than 1 millisecond and the position error was less than 0.5 mm. It was also confirmed that dose distributions reconstructed based on the log were in good agreement with measurements. A gamma index analysis with dose distribution measured with a 2-dimensional array detector and plastic phantoms loaded on a moving platform for a planar irradiation field resulted in a pass rate of 98% with dose-tolerance and distance-to-agreement levels of 1%, 1 mm, and 100% with 2%, 2 mm criteria. In the evaluation procedure, tumor motion is taken into account as a shift in the spot position on a static planning CT image, since the anatomical deformation due to tumor motion during irradiation is mitigated within the gating window, which is thought to be comparable or narrower than each phase of 4-dimensional CT (4DCT). We have evaluated the relationship between the phase of 4DCT and the gating window [29]. According to the evaluation, the movement of the marker within the gating window of ±2 mm was mostly within the expiratory phase of 4DCT (T50 or T60). The purpose of this study is to show the reliability of the dose distributions for liver tumors treated with RGPT by evaluating the accumulated dose distributions.

## MATERIALS AND METHODS

### A. Dose reconstruction method

**Fig. 1.**
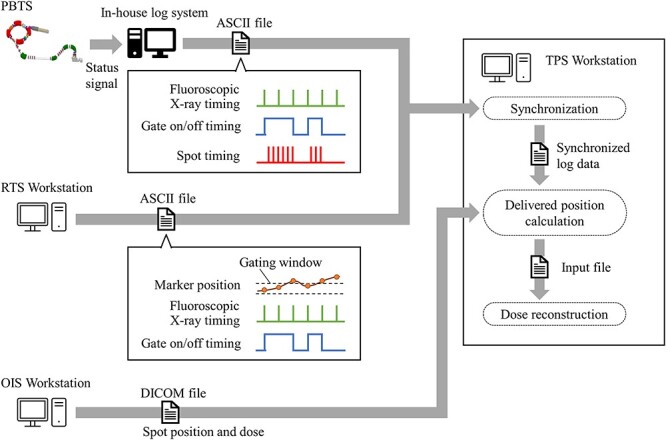
Simplified diagram of the procedure relating specifically to the log file generation and subsequent 3D dose reconstruction. Abbreviations: RTS = real-time tumor tracking system; PBTS = proton beam therapy system; OIS = oncology information system; TPS = treatment planning system.

The proton beam therapy system (PBTS) of Hokkaido University uses a synchrotron-based accelerator. Gated irradiation is performed on the basis of the measured position of fiducial markers placed near a tumor by two fluoroscopic X-ray imaging apparatuses installed in the gantry. Figure 1 shows the evaluation procedure of the log file generation and dose reconstruction. An in-house logging system, which consists of a workstation and an FPGA module (PXI-7842; National Instruments, Austin, TX) for signal measurements, it records the X-ray exposure timing, the gate timing, and the proton spot delivery timing by measuring the status signal of the power supply for the X-ray tube, the gating status signal, and the status signal of the RF extraction from the synchrotron, respectively. The PBTS log file is recorded in an ASCII log file and manually input to the treatment planning system (TPS) workstation. The real-time tumor tracking system (RTS) records the marker position, the X-ray exposure timing and the gate timing in an ASCII log file. The RTS log file records the exposure timing, the marker position recognized by the RTS, and the gate status for each X-ray exposure. The RTS log file is regularly stored on the TPS workstation by backup software on an in-hospital network. The RTS log file and PBTS log file are recorded in separate systems which are not synchronized. To acquire the position error of the marker from the planned position at delivery of each spot, a synchronized log data in ASCII format is generated from the RTS log file and PBTS log file on the basis of the X-ray exposure and gate timings by an in-house software in the TPS workstation. The position error of the marker from the planned position at delivery of each spot is calculated by interpolation of the marker position acquired at 33 millisecond intervals.

The spot positions and dose delivered at each spot are measured by monitors installed in the irradiation nozzle and are recorded in the oncology information system (OIS) as a log file in DICOM format. The OIS log file is regularly stored on the TPS workstation by backup software on the in-hospital network. The delivered position of each spot relative to the target is calculated according to equation (1) by regarding the change in the marker position as a spot position shift.

(1) \begin{equation*} R={R}_{OIS}-\Delta{R}_{RTS} \end{equation*}

Here, *R* is the delivered spot position, *R_OIS_* is the spot position recorded in the OIS, and Δ*R_RTS_* is the position error of the marker. As shown in equation (1), the influence of the movement of the marker within the gating window is dealt by shifting the spot position in the direction opposite to the tumor movement. The input file composed of the delivered spot positions and dose is generated for the dose calculation by the in-house software. The dose distribution of the treatment fraction is calculated in the TPS (VQA (Ver. 5.5.2), Hitachi) from the input file on a static planning CT image. Dose distributions for every fraction are summed up in the single planning CT.

### B. Clinical adaptation

#### Patient data

The study included 12 cases of 11 patients with liver tumors treated with RGPT [19, 30] from May 2016 to March 2017. We excluded four cases in a period when the log system for PBTS was not in operation.

CT images were taken at the end of the exhale phase with a 2.5-mm slice interval using a multi-detector CT scanner (Optima CT580 W; GE Healthcare, Waukesha, WI). The VQA treatment planning system was used for the treatment planning of RGPT, with a gating window size of ±2 mm in three directions (right–left, antero-posterior, and cranio-caudal). The window size was determined from the dose distribution and treatment time in previous studies [19, 29]. The clinical target volume (CTV) was delineated on the CT image by a radiation oncologist. Single-field uniform dose optimization was performed for all plans, and the CTV was set as the target. The lateral margin (calculated as the sum of the gating window size [2 mm], spot position error [2 mm], imaging-related error [1 mm]) and distal and proximal margins (3.5% of the proton range + 1 mm) were added to the CTV to address setup and range errors [31]. After the optimization, if the total monitor unit for a spot was greater than 0.04 (the maximum deliverable amount for a spot), the spot was split and rescanned until all of the monitor units were delivered [1]. The rescanning technique [32] that irradiates all spots multiple times slice-by-slice or volumetric rescanning is not utilized.

For four cases, the prescription dose was D99 = 76.0 Gy with a relative biological effectiveness (RBE) of 1.1 in 20 fractions. For the remaining four cases, the prescription dose was D99 = 72.6 Gy (RBE) in 22 fractions. Mean dose for normal liver (Liver—GTV) was required to be below 30 Gy (RBE) in all cases.

Volume of CTVs ranged from 17.5 to 649.3 cc. The spot intervals ranged from 5.0 to 7.0 mm, and its average was 5.5 mm. For three cases, the short-range applicator [33] was used for each field because the CTVs were in shallow regions. The log data were acquired in all 168 fractions for the eight cases. The delivered dose distributions were reconstructed on the basis of the log data.

### Analysis

The evaluation was performed for the maximum dose, minimum dose, D99 and D5–D95 for the CTVs and mean liver dose (MLD) scaled by the prescribed dose. These dosimetric parameters were compared between the planned dose distribution and the reconstructed dose distribution in terms of mean difference and 95% CI. Dosimetric parameters were also evaluated to determine whether the delivered dose distribution met the prescribed criteria for other OARs, e.g. stomach and bowel.

## Results

Log analysis results of marker displacement and spot position errors are shown in Table 1. Here, R_ALL_ is distance between marker position during the treatment and the planned position. The marker position was recorded throughout the treatment so that the position included information of marker motion both inside and outside the gating window. And R_SPOT_ is the distance between the marker position at spot delivery and the planned position. The marker positions at spot delivery were everywhere suppressed to the gating window size of 2 mm and the spot position errors were small relative to the marker positions.

**Table 1:** Log analysis results of marker displacement and spot position errors in RGPT; the values are given as the mean ± SD in millimeters

Case	R_ALL_	R_SPOT_	Spot position error
1	7.1 ± 8.9	1.2 ± 0.6	0.4 ± 0.2
2	11.0 ± 9.6	1.3 ± 0.8	0.4 ± 0.2
3	3.7 ± 3.2	0.9 ± 0.5	0.5 ± 0.3
4	3.1 ± 3.2	0.8 ± 0.5	0.4 ± 0.2
5	6.1 ± 5.9	0.9 ± 0.5	0.3 ± 0.2
6	4.1 ± 4.5	0.9 ± 0.6	0.4 ± 0.3
7	4.4 ± 4.6	0.9 ± 0.6	0.4 ± 0.2
8	4.6 ± 4.1	1.0 ± 0.6	0.5 ± 0.3

R_ALL_: Distance between the marker position during the treatment and the planned position,

R_SPOT_: Distance between the marker position at the spot delivery and the planned position,

SD: Standard deviation

**Fig. 2.**
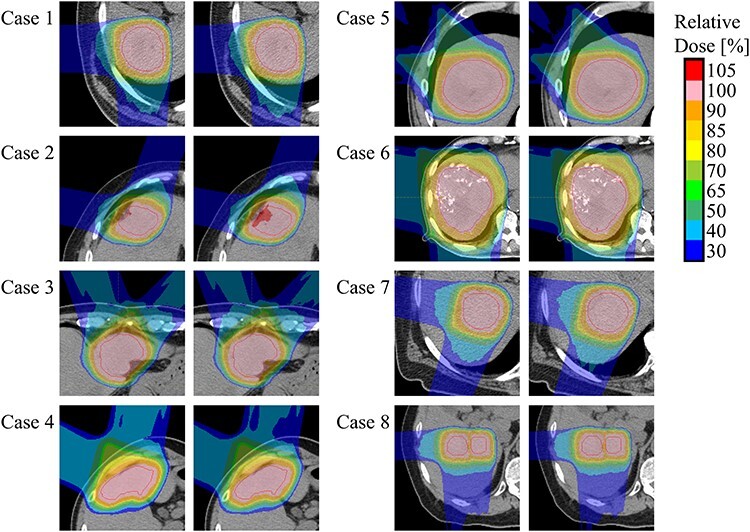
Dose distributions on an axial plane near an isocenter for eight cases. In each case, the left figures show planned dose distributions, and right figures show reconstructed dose distributions. The red contours indicate the CTVs.

Figure 2 shows an example of planned dose distributions and reconstructed dose distributions for the eight cases. The reconstructed dose distributions were in good agreement with the planned distributions.

**Fig. 3.**
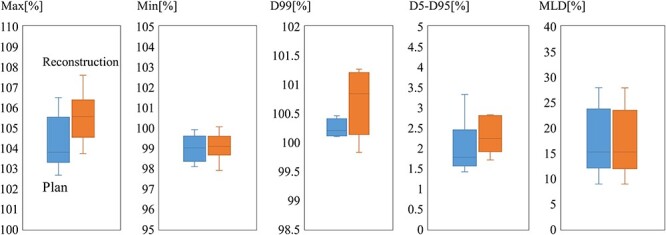
Box plots of maximum, minimum, D99, and D5–D95 for the CTV and mean dose for normal liver (MLD). Boxes shows the interquartile range from the first to the third quartile.

Figure 3 shows box plots of dose volume histogram (DVH) parameters for planned and reconstructed dose distributions. Regarding the maximum dose for the CTVs, the mean difference was 1.3% (95% CI: 0.6%—2.1%). The increase in maximum dose was at most 2.1%, which was of no clinical importance. Variations in the minimum dose for the CTVs were small. The mean difference of the minimum dose was 0.1% (95% CI: −0.5%—0.7%). Seven cases met the ICRU criteria in which the CTV dose should be from 95% to 107%. For the remaining one case, the maximum dose was 106.5% in the planned dose distribution, and the variation was +1.1%. The mean difference of the D99 was 0.4% (95% CI: −0.02%—0.9%). The MLDs of the delivered dose distributions were in good agreement with the planned. The mean difference of the MLD was −0.1% (95% CI: −0.2%—0.04%). The mean difference of the D5–D95 was 0.5% (95% CI: 0.2%—0.8%). The other DVH parameters not shown in Figure 3 met the prescribed criterion.

**Fig. 4.**
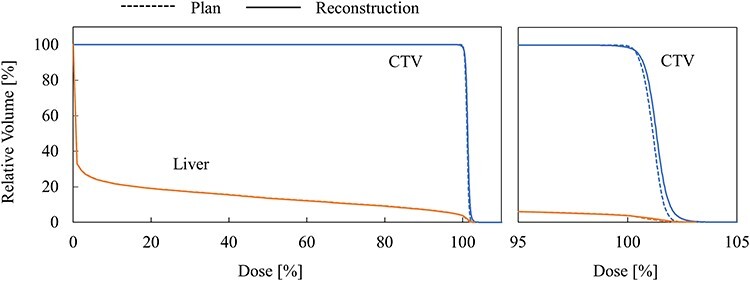
DVH comparison in case 1. The right figure shows enlarged view of DVH near 100% dose.

**Fig. 5.**
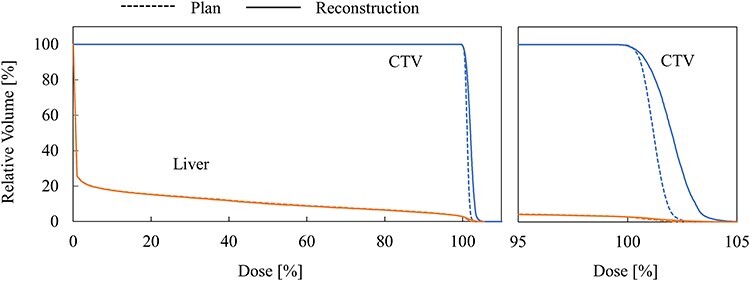
DVH comparison in case 3. The right figure shows enlarged view of DVH near 100% dose.

Figures 4 and 5 show DVH curves for two cases in which the variation between the planned and reconstructed dose distributions were large.

The DVH curve for case 1 is shown in Figure 4. In this case, maximum dose, minimum dose, D99, D5–D95, and MLD were + 3.0%, −1.5%, −0.3%, 0.3%, and − 0.1%, respectively. Some positions showed somewhat high or low doses, but the variation in uniformity evaluated with D5–D95 was small. The variations in D99 and MLD were small.

The DVH curve for case 3 is shown in Figure 5. In this case, maximum dose, minimum dose, D99, D5–D95, and MLD were + 2.2%, 0.0%, 0.0%, +1.2%, and − 0.2%, respectively. The variation in minimum dose was small, while the maximum dose and D5–D95 significantly increased. The variations in D99 and MLD were small.

**Fig. 6.**
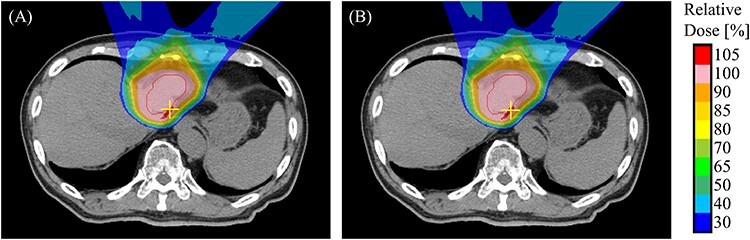
Hot spot in reconstructed dose distribution of case 3. The figure (a) shows the planned dose distribution, and the figure (b) shows the reconstructed dose distribution. The red contour and the cross mark indicate the CTV and the maximum dose point. This axial plane contains the maximum dose point of the CTV and is located at the head-wise end of the CTV.

Figure 6 shows the maximum dose point in the reconstructed dose distribution for case 3. The maximum dose point is indicated by the cross mark. This point is also in a high-dose region in the planned dose distribution. The point is located on the side of the distal end region in both irradiation fields, and in this vicinity the dose at each spot is large; this makes changes in the dose distribution due to the marker displacement and the spot position errors are more likely to occur here than in other regions.

## DISCUSSION

This study showed the reliability of the dose distributions for liver tumors treated with RGPT by evaluating the accumulated dose distributions. The dose distributions were calculated on the basis of log data of tumor motion and log data of beam delivery that included timing information of the spot irradiations.

Kanehira *et al.* [29] calculated dose distributions for four different start phases of respiration. However, the present method does not require such a calculation because it reconstructs the dose distributions on the basis of actual timing data.

Colvill *et al.* [27] proposed a dose reconstruction method incorporating beam’s-eye-view tumor motion by shifting each spot in the opposite direction of the tumor and in-depth motion as the beam energy changes for each spot with simulated proton spot delivery durations by assuming accelerator operation. In our study, the timing data of beam delivery acquired from an accelerator system are synchronized to log data of tumor motion so that a more precise dose distribution can be reconstructed.

We statistically analyzed DVH parameters for eight cases with liver tumors treated with RGPT. The maximum dose in the CTVs increased, with a mean difference of 1.4%. The increase in maximum dose was at most 2.1%, which was of no clinical importance. The mean difference in the minimum dose, D99, and D5–D95 in the CTVs and the MLD were below 1%. This indicated that dose distortion was suppressed by gated irradiation. The rescanning technique [32] was not utilized in the RGPT, spots requiring large irradiation amounts are irradiated multiple times and so interplay effects would be mitigated.

As shown in Fig. 6, maximum dose spots were on the side of the distal end region. As the dose of each spot was large, the dose distribution was sensitive to the spot position and target position. The maximum dose increased because of the hot spot generated in the CTV. The small change in the minimum dose and the increase in the maximum dose reduced the uniformity of the dose distribution, leading to an increase in D5–D95.

**Fig. 7.**
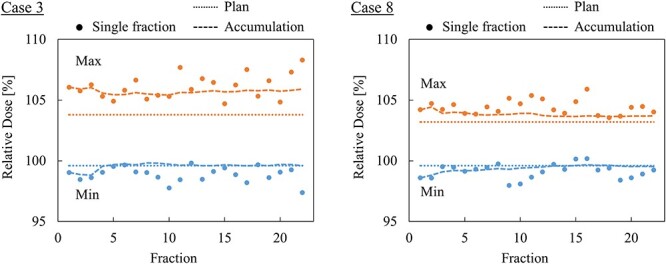
Maximum dose and minimum dose in CTV for cases 3 and 8.

Figure 7 shows the maximum dose and minimum dose through all fractions for two cases in which the variation between the planned and reconstructed dose distributions were large (case 3) and small (case 8). In both cases, the minimum dose in the accumulated dose distribution converged to the planned value as the fraction increased. This suggests that the positions of the cold spots were randomized through the fractions such that the accumulated minimum dose increased. In case 3, the maximum dose did not converge by accumulation. On the other hand, the accumulated maximum dose converged to the planned value as the fraction increased in case 8. The mean error (standard deviation) of the delivered spot position, i.e. *R* in equation (1), was 1.21 mm (0.62 mm) in case 3 and 1.24 mm (0.56 mm) in case 8. A positive correlation with the maximum dose and the standard deviation of the delivered spot position error was observed in case 8, but no correlation was observed in case 3. The absence in convergence of the maximum dose in case 3 indicates that the location of the hot spot was reproduced through the fractions such that the accumulated maximum dose did not decrease. As shown in Figure 6, the hot spot in case 3 located at a position where the distal ends of the two irradiation fields overlap, so that the spots contributing to the hot spot generation were small in proportion to the whole. This suggests the validity to consider that there was no correlation between the maximum dose and the standard deviation of the delivered spot position error. The reproducibility of the hot spots might be decreased by adjusting beam angle of irradiation fields to avoid overlapping of distal end regions.

In this study, dose distributions of SSPT were validated by dose reconstruction based on the log files. Dose validation after every fraction as post-treatment Quality Assurance (QA) can increase the reliability of the dose distribution through the whole of the treatment duration. Evaluation of reconstructed and accumulated dose distributions provides valuable information to support decisions on replanning for an adaptive therapy.

In SSPT, dose reconstruction is easier to realize than a broad beam proton therapy because a log file records spot positions and doses. In this study, since common timing data existed in the RTS log and PBTS log, the synchronization could be realized. If the original log data are synchronized, this procedure would not be necessary. The log-based dose reconstruction function should be provided by the vendor.

As the delivered dose distributions were reconstructed on the static planning CT, setup errors and inter-fractional deformations were not considered in the calculations of the accumulated dose distribution. This is a major limitation of the study. In our institute, however, every fraction is irradiated after ensuring that the changes in the positional relationship between the marker and the bone structure (and surrounding organs) is within the tolerance determined by the particular robustness evaluation for every patient in the treatment planning process. Therefore, it is considered that setup errors and anatomical deformation are suppressed to the extent that it does not significantly affect the dose distribution. If a daily CT scan is available, this could improve the accuracy of the dose reconstruction. Regarding intra-fractional deformation, in this study, tumor motion was regarded as a spot position shift under the assumption that the anatomical deformation due to tumor motion during irradiation is mitigated by gating in RGPT. It is a limitation of this study in evaluating the interplay effect that the reconstruction is based on a static CT image. For more accurate evaluation, it would be better to reconstruct dose distribution based on an ideal 4D image with a high time resolution. It is possible to evaluate the effects of anatomical changes in the dose distribution by utilizing 4DCT images. However, the results might be the same even when considering anatomical changes utilizing currently available 4DCT because the gating window size of ±2 mm is sufficiently small compared with the marker motion in one phase of the 4DCT images.

In the method of this study, the dose distribution is reconstructed on the basis of the spot position, dose, and marker position by assuming that the shape of the spot does not change. However, in an actual irradiation, the spot size slightly changes for each spot. The change in the spot size varies depending on the energy and number of irradiation spots, and the typical change is about 0.3 mm, which is considered to be negligible with respect to the reconstruction of the dose distribution. To reconstruct the dose distribution by taking into consideration the size change per spot, it is possible to calculate the fluence distribution based on the spot shape, as reported by Furukawa *et al.* [34].

In this study, the reconstruction of the dose distribution was limited to consideration of the spot dose error and spot position error perpendicular to the beam axis. The spot position error parallel to the beam axis was not taken account when reconstructing the dose distribution. Colvill *et al.* [27] proposed the dose reconstruction method incorporating beam’s-eye-view tumor motion by shifting each spot in the opposite direction of the tumor and in-depth motion as beam energy changes for each spot.

Finally, the conclusions of this study are limited by the small number of patients. Further comparative clinical data are needed.

## CONCLUSION

The evaluation of the accumulated dose distributions reconstructed by utilizing log data on tumor motion and spot information indicated the reliability of the dose distributions for liver tumors treated with RGPT-SSPT. A positive correlation with the maximum dose in CTV and the delivered spot position error was observed in a case where the accumulated maximum dose converged to the planned value as the fraction increased, but no correlation was observed in a case where the maximum dose did not converge by accumulation.

## FUNDING

This work was supported by AMED under Grant Number JP 19he1602004.

## Notes

COI: Four of the authors (Takahiro Yamad, Hideaki Nihongi, Yusuke Fujii and Shusuke Hirayama) are the employees of Hitachi Ltd., Tokyo, Japan.
